# Caregivers' Experiences With Involuntary Oral Care for Individuals With Dementia Within the Dutch Care and Coercion Act

**DOI:** 10.1111/ger.70019

**Published:** 2025-09-19

**Authors:** Maud Jonker, Coos Engelsma, David J. Manton, Anita Visser

**Affiliations:** ^1^ Department of Gerodontology, Center for Dentistry and Oral Hygiene University Medical Center Groningen, University of Groningen Groningen the Netherlands; ^2^ Medical Ethics and Decision Making, Department of Ethics, Center for Dentistry and Oral Hygiene University Medical Center Groningen, University of Groningen Groningen the Netherlands; ^3^ Department of Cariology, Center for Dentistry and Oral Hygiene University Medical Center Groningen, University of Groningen Groningen the Netherlands; ^4^ Department of Paediatric Dentistry, Academic Centre for Dentistry Amsterdam (ACTA) Vrije Universiteit Amsterdam and University of Amsterdam Amsterdam the Netherlands

**Keywords:** care‐resistant behaviour, dementia, involuntary oral care, legislation, oral health care

## Abstract

**Background:**

Individuals with dementia frequently show care‐resistant behaviour toward the provision of oral care. Providing oral care despite care‐resistant behaviour is legally considered to be involuntary oral care. In the Netherlands, the provision of involuntary oral care to incapacitated individuals with dementia is regulated by the Care and Coercion Act (CCA) since 2020.

**Objective:**

This study aimed to assess how care providers experienced the CCA in the context of involuntary oral care for individuals with dementia.

**Methods:**

32 one‐on‐one semi‐structured interviews were conducted with different care providers involved in oral care for incapacitated individuals with dementia.

**Results:**

Through the interviews, multiple experiences concerning the CCA were identified. These experiences were related to: (1) awareness of involuntary care, (2) implementation of the law, (3) definitions, (4) circumvention of the law and (5) responsibility for involuntary care.

**Conclusion:**

The CCA creates awareness about involuntary oral care among care providers. However, many care providers view the legal definitions differently, causing variation in the interpretations and applications of the legislation. Also, several care providers feel that the law could result in quicker discontinuation of oral care provision. Care organisations and policymakers should provide clarity concerning the legal definitions and provide training on legislation and the provision of oral health care in cases of care‐resistant behaviour.

## 1 Introduction

Presently, an increasing number of older individuals with dementia still retain their natural dentition and often require assistance in oral care to maintain adequate oral health [1, 2]. Unfortunately, incapacitated individuals with dementia frequently show resistant behaviour toward oral care provision, as they no longer understand the intentions of others to provide oral care [3, 4]. This care‐resistant behaviour toward oral care provision often leads to difficult ethical dilemmas. Without adequate assistance, the individual's oral health deteriorates, often causing pain and infections, which can influence their health and quality of life [5]. Poor oral health has been linked to several general health conditions, including pneumonia, compromised diabetic control, arthritis and endocarditis [6].

In case discontinuing oral care leads to a risk of serious harm to an individual who lacks the mental capacity to make decisions about such care, it may be necessary to provide care involuntarily. Involuntary oral care refers to the administration of oral care without the consent of the patient [7]. Involuntary oral care should be minimised, since it can have a negative psychological impact on individuals, often leading to feelings of anger, humiliation, fear or depression, and it can also cause moral distress among caregivers [8].

Therefore, in several countries, the provision of involuntary oral care is regulated by law. For instance, in Norway, the provision of involuntary oral care is regulated by the Health and Care Services Act, and in the United States, it is regulated by the Mental Capacity Act [9, 10].

In the Netherlands, involuntary oral care is regulated by two laws, the Care and Coercion Act (hereafter: CCA; Dutch: Wet zorg en dwang) and the Compulsory Mental Healthcare Act (Dutch: Wet verplichte geestelijke gezondheidszorg). These laws were implemented in 2020, replacing the Special Admissions to Psychiatric Hospitals Act (SAPHA; Dutch: Wet bijzondere opnemingen in psychiatrische ziekenhuizen) [11].

The CCA regulates involuntary oral care in home care and nursing home settings for incapacitated individuals with an intellectual disability and individuals with a psychogeriatric condition (such as dementia). It applies to individuals lacking the mental capacity to make their own decisions about the proposed care. Also, involuntary oral care is legally impermissible for individuals with an intellectual disability or with a psychogeriatric condition who retain decision‐making capacity concerning oral care [7].

The core principle of the CCA is ‘No, unless…’, which means that providing involuntary oral care is not allowed, unless not providing such care leads to serious harm (or fails to remove serious harm). When an incapacitated individual with, for example, dementia shows care‐resistant behaviour, the CCA stipulates that care providers seek out alternative ways to administer care voluntarily. If these attempts fail, and not providing care will (potentially) lead to serious harm, care providers are required to follow a step‐by‐step procedure [2]. One of the steps involves organising a multidisciplinary meeting (MDM) with different care providers in the relevant medical field. The goal is to assess (i) the risk of serious harm and (ii) whether the provision of involuntary care is the last remaining option to prevent harm. If care providers reach consensus that there is a high risk of serious harm when the intended care is not provided, and that involuntary care is the last remaining option, involuntary care is allowed when it meets the following three criteria: (1) proportionality: the proposed treatment is in reasonable proportion to the purpose of its application, (2) subsidiarity: the least invasive treatment is chosen, and (3) effectivity: the proposed treatment achieves the intended goal and lasts no longer than necessary [7].

A recent study of Jonker et al. (2024) [12] reported that many oral care providers of individuals with dementia lack knowledge about the CCA [12]. Similarly, a report by the Dutch Health and Youth Care Inspectorate from 2021 states that care providers lack knowledge about the CCA and its practical implementation [13]. This lack of knowledge increases the risk that involuntary (oral) care is provided to individuals with dementia without the careful considerations and procedural steps required by law [13]. The lack of knowledge could also result in quicker discontinuation of care in cases of care‐resistant behaviour, driven by the misconception that involuntary (oral) care provision is never allowed [12].

In addition, many care providers lack knowledge about the importance of oral care, making it possible that they do not consider oral health problems as serious harm [14, 15]. As a result, even with sufficient legal knowledge and a conscientious effort to follow the law, the option to provide involuntary oral care may often not be considered.

To gain more insight into legislation regulating involuntary oral care, the aim of this study was to assess how care providers experience the CCA in the context of involuntary oral care for individuals with dementia. To achieve this aim, the study addressed the following research question: ‘What are the experiences of caregivers with the implementation and execution of the CCA in involuntary oral care provision for individuals with dementia?’

## 2 Materials and Methods

### 2.1 Study Design, Sample Size, Setting and Participants

To identify care providers' experiences with the CCA, semi‐structured one‐on‐one interviews were conducted with professional care providers involved in oral care for incapacitated individuals with dementia residing at home or in nursing homes in the Netherlands. The interviews were conducted between December 2023 and October 2024 [16].

The inclusion criterion for participants was being a registered healthcare provider involved in decision‐making about, or provision of, involuntary oral care to incapacitated individuals with dementia who show care‐resistant behaviour in the Netherlands.

The sample size was based on data saturation. Therefore, the study responses were monitored by two researchers (M.J. and C.E.) to identify when data saturation occurred. This occurred when M.J. and C.E. agreed that three consecutive interviews did not provide any new insights.

### 2.2 Recruitment Procedure

The recruitment procedure was based on creating a maximum variation sample size, using purposive sampling. Individuals from different ages, sexes, occupations and provinces in the Netherlands were sought to participate. Potential participants were approached through several methods, including the authors' network, participant lists of a geriatric sedation education programme, and targeted outreach via social media to specific care providers [16].

### 2.3 Participant Information and Informed Consent

Before the interview, participants received study information, including an informed consent form, by e‐mail. Hereby, participants were informed that the interview would be recorded and that the results would be made completely anonymous. Next, the interviews were conducted via video calls (Microsoft Teams; Microsoft Corp., WA, USA) by one researcher (M.J.) Before the interview recording started, participant consent was confirmed. During the interview, an interview guide (Table 1) was used as a flexible guideline to allow expansion on certain topics or comments by the participant. Afterwards, all interviews were transcribed verbatim by one researcher (M.J.).

**TABLE 1:** Summary of semi‐structured interview guide [16].

1. What is your work function? How long have you been doing this job?
2. What does a working day look like for you?
3. In what way are you involved in the oral care of residents?
4. Do you ever deal with residents who show care‐resistant behaviour toward oral care? What do you do in such cases?
5. What are your experiences with providing involuntary oral care? Can you give examples? Do you encounter certain issues (barriers) concerning involuntary oral care? What would make it smoother for you to perform involuntary oral care (facilitators)?
6. What do you consider reasons to provide involuntary oral care as a last resort? What is your opinion concerning involuntary tooth brushing?
7. What do you consider involuntary care? Can you give examples?
8. Does the Care and Coercion Act play a role for you in providing involuntary oral care? How so?
9. What is your age, what is your gender, and in what province do you work?

Prior to data collection, two pilot interviews were conducted to test the interview guide [16].

### 2.4 Data Analysis

The results were qualitatively analysed by using thematic analysis according to Braun and Clarke [17]. All interviews were previously transcribed by one researcher (M.J.) and were uploaded in Atlas.ti (version 9.13.2, Scientific Software Development GmbH, Germany), which was used to code the transcripts. Two researchers (M.J. and C.E.) independently coded the transcripts ‘line‐by‐line’, and subsequently created categories and themes. Lastly, they discussed and reached consensus about the categories, and developed themes together.

### 2.5 Researcher Positionality and Reflexivity

The interviews were conducted by one researcher (M.J.), a dentist with specific training in qualitative healthcare research and experience in geriatric dentistry. The dental professionals who participated in the study were aware of M.J.'s occupation. However, since they worked in the same field, this was not expected to influence their response. For all other participants, M.J.'s occupation was either not disclosed or, if asked, only revealed after the interview, to prevent this from influencing participants' responses. The second researcher (C.E.), an experienced researcher and lecturer in Ethics with no dental clinical background, collaborated closely with M.J. through peer debriefing to prevent individual bias and enhance a balanced interpretation of the data.

## 3 Results

In total, 32 semi‐structured interviews were conducted, including eight geriatric‐focused dentists, three geriatric‐focused dental hygienists, six carers, seven nurses, three geriatric specialists, two dementia case managers and three general medical practitioners. The interviews lasted between 19 and 46 min. Four participants were male and 28 were female, ages ranged from 27 to 79 years, and workplaces were spread across the Netherlands. The dementia case managers and general medical practitioners were specifically involved in oral care in home care settings.

**FIGURE 1:**
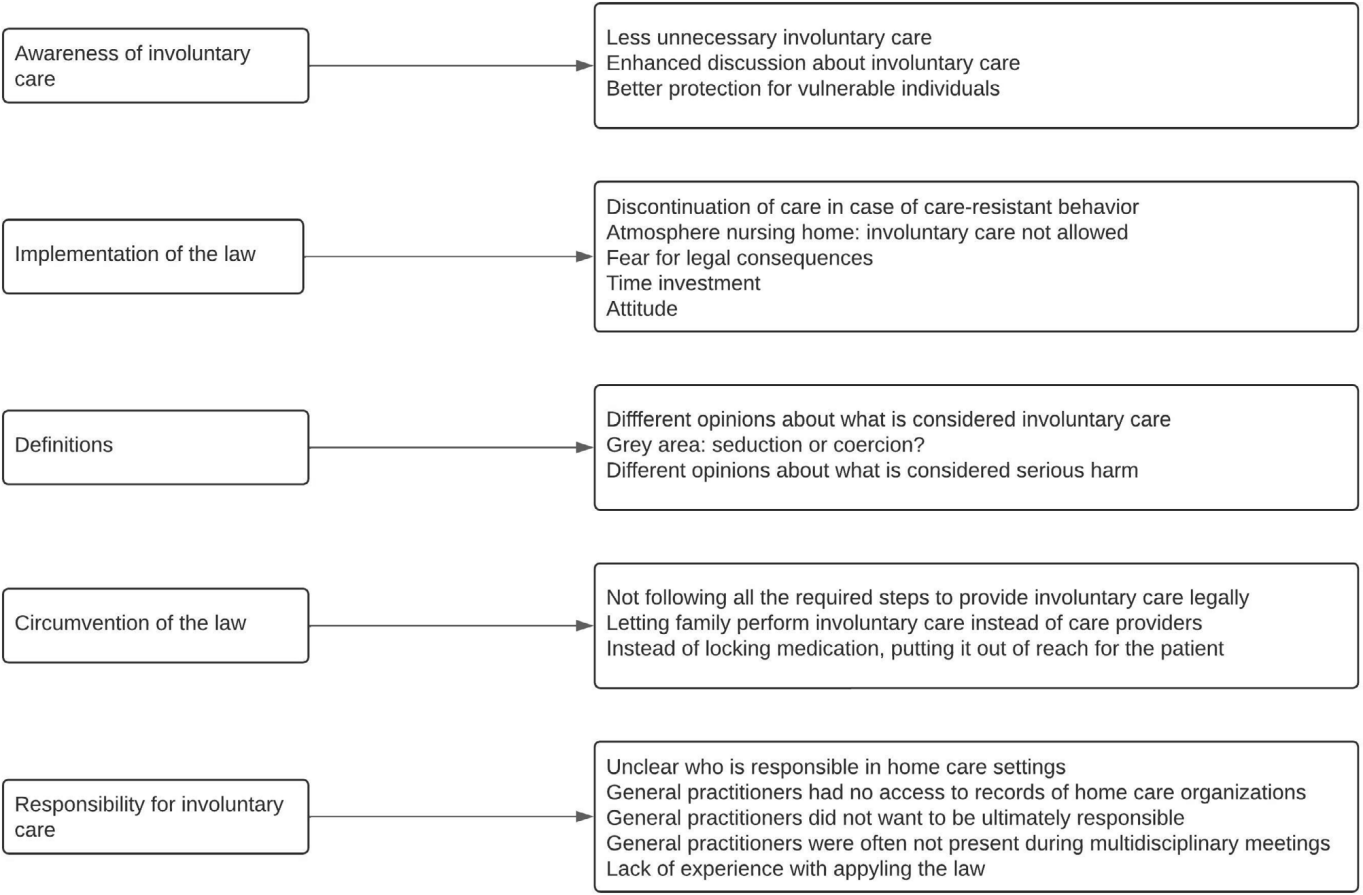
Overview of identified themes concerning experiences of Care and Coercion Act.

Multiple experiences concerning the CCA and oral care were identified through the interviews and were divided into five themes: (1) awareness of involuntary care, (2) implementation of the law, (3) definitions, (4) circumvention of the law, and (5) responsibility for involuntary oral care. An overview of the themes is given in Figure 1. The themes are illustrated by quotes extracted from the interviews. As the interviews were held in Dutch, these quotes were translated to English. The quotes were back‐translated to preserve their original meaning, and they were minimally adjusted to improve readability without altering the participants' intended message.

### 3.1 Awareness of Involuntary Care

Several participants reported that the law enhanced discussion concerning involuntary (oral) care, and thereby increased awareness concerning what counts as involuntary (oral) care and what care is necessary to provide. This increased awareness could decrease the unnecessary provision of involuntary (oral) care and stimulate the search for voluntary alternatives.

…it's about raising awareness, that law, as far as I'm concerned, is a guideline to safeguard people's autonomy for as long as possible, and for us to understand why they don't do certain things. Is it because of their illness, their personality, or life events they've experienced? That law helps you think in that way. (Geriatric specialist, Interview 3)

That is the advantage of such a law: it forces you to think about what constitutes coercion. […] Is this really necessary, and are there any alternatives? And if there aren't, is this the least restrictive measure I can apply? (Geriatric specialist, Interview 1)

### 3.2 Implementation of the Law

Multiple participants thought that oral care is discontinued quicker due to the CCA. In cases of resistance, they said, care providers did not search for alternatives to provide oral care voluntarily but used the law as an excuse to justify not providing oral care.

…Healthcare workers often don't particularly enjoy providing oral care for someone, and then [after implementation of the law] it becomes even easier to say, ‘Well, the person doesn't want it, so we'll just stop doing it.’ (Dental Hygienist, Interview 3)

But the caregivers often also say, ‘She doesn't want to.' […] so sometimes I get the feeling that they find it a bit easy to say, 'Yeah, she doesn't want to, so I can't do anything because of the Care and Coercion Act’. (Dentist, Interview 5)

Another potential reason why oral care in cases of care‐resistant behaviour is discontinued quicker due to the CCA is that some nursing homes created an atmosphere that involuntary care in general is not allowed. As a result, care providers could feel a fear of legal consequences if they tried to provide care to an individual who shows resistance.

I still remember when this [the CCA] just started, and we gave trainings, that they [care providers] all said, ‘We're not allowed to do anything anymore’ […]. And I really thought, ‘Oh dear, from the organization it's being imposed so heavily, watch out.’ And I think they just think, ‘Oh no, soon I'll get called out if I do something that's not right.’ (Dental Hygienist, Interview 3)

Furthermore, multiple care providers found that the CCA increased their administrative burden.

I kind of dislike it when there's a lot of administrative hassle around it. (Dentist, Interview 2)

…that Care and Coercion Act is nothing but a huge disaster that has come over us. […] Oh, it's all those rules that you have to follow, step‐by‐step plans with phases, involving other people. Trying to create objectivity with that, while it's completely unnecessary. (Geriatric specialist, Interview 1)

In addition, a geriatric specialist expressed general dissatisfaction with the implementation of the new law, and particularly with the alleged distrust toward care providers involved by it:

It's a law based on distrust and not on the trust of the caregiver. And, it's also a law that was already flawed when it was introduced in 2020, so it needed improvements every three months. It's a law that claims to protect vulnerable people, but it was already completely wrong from the start. (Geriatric specialist, Interview 1)

Not all care providers experienced the law's obligatory steps in such a negative way. Some praised its systematic approach and said it did not have to be very time consuming.

…I do feel the freedom to choose whether or not to take certain actions within this law. It's about a systematic approach to thinking, ensuring that you don't just act impulsively. So, I—we—can work with this just fine. (Geriatric specialist, Interview 3)

… these are often people where, even before I'm involved, the Care and Coercion Act has already been applied more frequently for other reasons. So those procedures then go very easily and quickly, because everyone immediately agrees, and there has already been deliberation. (Dentist, Interview 4)

### 3.3 Definitions

During the interviews it became clear that how ‘involuntary care’ is defined varies among participants.

We should not provide involuntary care, but what exactly is involuntary care? That line is still not very clear to everyone. (Nurse, Interview 3)

To give examples of the unclear definition of involuntary care, participants were presented with a scenario involving an older individual with dementia who resists tooth brushing by pressing their lips together. They were asked whether stimulation with the fingers or the toothbrush over the lips qualifies as involuntary oral care. Some participants did not consider this involuntary care, but rather a way of communication:

No, I think that it is coercion when you need to restrain someone with two people to provide oral care. Well, that simply isn't acceptable. But I don't think it's coercion when you try to use the toothbrush in every possible way. (Nurse, Interview 2)

No, not that, because you're actually stimulating their reflex to open their mouth. (Carer, Interview 5)

Contrarily, other participants did consider stimulation of the lips a form of involuntary oral care:

I do consider that involuntary care because someone clearly indicates at the start, by keeping their lips tightly closed, ‘I don't want this’. When a staff member continues to push, rubbing over the lips, and the person eventually opens their lips, I consider that involuntary. (Dementia case manager, Interview 2)

Another example possibly qualifying as involuntary care is holding hands. Whether holding hands during oral care provision qualifies as involuntary care is also something that is unclear for some participants:

How far do you go with coercion? What does the Care and Coercion Act really entail? It's a bit of a broad concept. […] You're not allowed to put the brake on a wheelchair, but if, for example, you hold someone's hands while brushing their teeth, is that coercion as well? (Carer, Interview 2)

Some participants said that holding hands while brushing teeth is involuntary oral care and that it should be discussed with colleagues:

Just standing behind someone for a moment, holding their hands. That too is part of the Care and Coercion Act; you are restricting someone's freedom, so you need to think about that. (Geriatric specialist, Interview 3)

Contrarily, others said that whether holding hands constitutes involuntary care depends on the reaction of the patient, and that it could also be a form of contact. To illustrate, a carer answered the following to the question whether holding hands is involuntary care:

No, because actually, you can be very creative. An important thing in this, in everything, is that you really connect with them, because when you are in contact and connected with the resident, the resident will feel safe more quickly than if you just come in and say, ‘Well, open your mouth, I want to brush.’ That just doesn't work, so in this situation, it's really important that you make and maintain contact. (Carer, Interview 5)

In addition to the unclear definitions of ‘involuntary care’ and ‘coercion’, participants were also uncertain about what counts as care‐resistant behaviour. They experienced a grey area between persuasion and coercion.

…If I can sit next to her [the patient] for 15 min, rub her hand a little, and have a nice chat, then after 20 min, I can just look in her mouth without anyone saying ‘No’. Is that coercion? Or is that persuasion? Or what is that? […] Is it allowed or not? One doctor thinks it's not. They say: ‘Once “no” remains “no”’. And another doctor says, ‘Oh, then it's fine because you're not really forcing’. But that, of course, makes it quite difficult. (Dentist, Interview 7)

Where do you draw the line with coercion, and where do you draw the line with pushing a little to get things done? That is also very subjective. (Dentist, Interview 2)

Participants also had divergent opinions about what counts as serious harm (possibly justifying involuntary care). For instance, while a dental hygienist considered not brushing an individual's teeth for a year as serious harm, a geriatric specialist disagreed.

What is a serious harm? Well, if you don't brush for a year. (Dental Hygienist, Interview 3)

Participant: I think, a set of teeth that deteriorates, well, that's okay in a year.

Interviewer: So you're saying that it doesn't actually need to be brushed if someone has a year to live?

Participant: It doesn't need to be, no. (Geriatric specialist, Interview 1)

### 3.4 Circumvention of the Law

Multiple participants described complying with the law as quite complicated, especially in home care. As a result, care providers often resort to various tricks to circumvent the law. A general example of this is the following:

You're not allowed to put medication in the locked medication cabinet, for example, because then the clients can't access it. But if you place it somewhere higher, then technically the client can still reach it. So, in that way, you kind of circumvent the rules of the Care and Compulsion Act. (Dementia case manager, Interview 1)

Also, because the law is applied less frequently in home care settings compared to nursing homes, care providers often lack experience with its implementation. Due to their lack of experience, care providers were more inclined to circumvent the law rather than to apply it.

It's something that rarely occurs [enforcing the CCA in home care settings], so it's not that we easily think, “Oh, you should do it this way or that way.” I always have to guess as well, and that takes extra time. So, I understand that it's easier to circumvent the rules. (General practitioner, Interview 2)

Furthermore, a dentist expressed that the legally required steps are not always taken before involuntary oral care is administered:

…look, even holding hands isn't officially allowed but sometimes it works. And then to initiate an entire procedure for care and coercion, while, for example, if there's a family member present who can just holds the mother's hands for a moment. After which I can calmly observe, you often see that once you get started, they're perfectly fine with it, then you can let go of the hands. Often, it's just that initial moment when you're about to go into the mouth. So I would still say that officially it might not be entirely in line with the rules, but I also believe that in practice, the effect it has and the limited impact of the compulsion on the person is something I can live with. So, it's just a matter of briefly refusing and then allowing it, and I can do a check or just use a toothbrush in the mouth. (Dentist, Interview 4)

This example illustrates an approach care providers use to avoid following the law well: letting the patient's family perform involuntary care, as the CCA does not apply to them. As a dementia case manager stated:

…that [use of bed rail] also restricts freedom of movement, but the partner, for example, is allowed to raise the bed rail, while we are not permitted to do so. (Dementia case manager, Interview 1)

### 3.5 Responsibility for Involuntary Care

Participants highlighted several issues regarding responsibility, mostly in home care situations. While the geriatric specialist is ultimately responsible for a patient's care in nursing homes, in home care settings it is frequently unclear who is ultimately responsible: the general practitioner or the geriatric specialist.

It's still a very complicated matter, because who is ultimately responsible? When you're talking about the Care and Coercion Act, is that the general practitioner, or should it be a geriatric specialist? (Dementia case manager, Interview 2)

In addition, a general practitioner also stated that they did not want to be ultimately responsible.

…no, actually, I really wouldn't like that, because you can already see how many people I'm responsible for in terms of care, quality, their education, and their collaboration. No, I really don't want to add more to that. (General practitioner, Interview 2)

Noting that the general practitioner is ultimately responsible, a dementia case manager experienced multiple obstacles regarding the execution of the CCA:

… a big obstacle in home care, is that the general practitioner is the one who is ultimately responsible. But the general practitioner can't access the files of the home care organization. And the general practitioner is also super busy, so they really don't want to be responsible for everything that home care does. […] Sometimes you have a multidisciplinary meeting at the general practice. But the general practitioner isn't always physically present, often it's the practice nurse who joins instead. (Dementia case manager, Interview 1)

These obstacles are similar to the obstacles experienced by general practitioners. They also mentioned a lack of time, not having access to the files of the home care organisation, and not even wanting access to those files. Furthermore, all general practitioners described limited to no experience with the CCA.

We have 53 home care organizations. 53, I mean, with every new clinic they set up, I have to deal with yet another referral system. I really don't want to be involved in that. It's driving us crazy, and what would I have to do then? I would have to start reading how they've done things, what they've done. So no, I really wouldn't want that, because it gives the impression that I could keep up with it or manage it, and I really don't need that. (General practitioner, Interview 2)

## 4 Discussion

This paper describes how Dutch legislation regulating involuntary oral care for individuals with dementia is experienced by care providers who are involved in the oral care provision to individuals with dementia. An important finding is the variety of care providers' interpretations of the concepts of care‐resistant behaviour, involuntary oral care, and serious harm.

The law states that care must be discontinued in cases of care‐resistant behaviour, and that a specific procedure must be followed if there is potential for serious harm [7]. However, variation was revealed in how care providers interpreted care‐resistant behaviour and how they applied these rules. Some care providers would immediately stop care in case of brief verbal resistance (such as saying ‘No’ once), considering this care‐resistant behaviour, and continuing any form of care as involuntary care. Others did not consider continuing care as involuntary care and chose to explore alternative options, such as distraction techniques (e.g., different timing or help of a loved one), to try to provide care voluntarily. A potential reason for this difference in interpretation is that the CCA itself does not define the concept of ‘care‐resistant behaviour’ [7]. The Dutch Society for Oral Health Care Guidelines (KIMO) and the Dutch Association of Elderly Care Physicians (Verenso) have tried to clarify this concept [18, 19]. However, they do not provide clear practical examples, leaving its interpretation largely up to individual care providers. Therefore, specific training with practical examples by policymakers could potentially reduce the difference in interpretation among care providers.

The guideline by Verenso clarifies that resistance is leading and should be respected. However, not all brief behavioural changes need to be considered care‐resistant behaviour, nor does this imply that care must be discontinued entirely. Sometimes care can be provided voluntarily at a later moment, without needing to follow the step‐by‐step procedure of the CCA [19]. If all voluntary attempts are unsuccessful, the steps of the CCA must be followed.

Interestingly, our study found multiple interpretations of what care is considered involuntary. Some care providers interpreted stimulation of a patient's lips as involuntary oral care, while others did not. Similarly, opinions varied regarding holding hands: some considered this involuntary oral care, while others did not. Hence, there is no agreement among participants on what counts as involuntary oral care. Consistent with this finding is a report by the Dutch Health and Youth Care Inspectorate from 2024, which revealed that care providers are sometimes insufficiently aware of what constitutes involuntary care [20].

Furthermore, divergent opinions were revealed about what oral problems are considered to result in serious harm. For instance, a dental hygienist perceived not brushing an individual's teeth for a year as serious harm, whilst a geriatric specialist disagreed. This difference in opinion is not surprising, as oral health care is barely discussed during the Dutch education program for medical staff [16]. The variation in what oral problems are considered serious harm is also confirmed by Jonker et al. (2024) [12], who showed that oral health problems were acknowledged as serious harm more often by dental professionals than by non‐dental professionals [12]. This highlights the importance of training concerning the consequences of poor oral health.

When non‐dental care providers do not consider oral care problems as (potentially) seriously harmful, they will read the law as allowing them to discontinue care. However, this can well have rather worrying consequences. As described in the introduction, discontinuing oral care will lead to poor oral health, which is associated with multiple general health diseases, such as cardiovascular diseases, respiratory infections, and complications in regulating diabetes [6]. Also, poor oral health will increase the risk of dental caries, which can cause broken teeth, chewing problems, pain and infections, ultimately impacting an individual's quality of life [21, 22]. In addition, neglecting oral hygiene for two weeks will already result in gingivitis, causing infection [23]. If gingivitis is left untreated, it may progress to periodontitis, which will ultimately result in tooth loss [24].

Another significant finding was that participants felt that oral care was discontinued quicker since the implementation of the CCA. Two potential causes for the quicker discontinuation of oral care were described.

Firstly, some care providers felt the law was used as an excuse to discontinue oral care in cases of care‐resistant behaviour, potentially because they were not particularly fond of providing oral care in the first place. This general dislike of care providers to provide oral care was also described by Hoben et al. (2017) [14] as a barrier to providing oral care to nursing home residents [14]. In addition, Willumsen et al. (2012) [25] and Weening‐Verbree et al. (2021) [26] found that approximately one‐tenth of nursing staff considered daily oral care provision an unpleasant task [25, 26].

Secondly, some care providers reported they feared legal consequences, as some nursing homes created an atmosphere that involuntary care was not allowed. A potential reason for this fear could be a lack of knowledge about the implementation of the law, which is confirmed by a report by the Dutch Health and Youth Care Inspectorate from 2021 and the study of Jonker et al. (2024) [12, 13]. The lack of knowledge about the CCA and its implementation is supported by the finding of the present study that some care providers (allegedly) chose to discontinue care rather than discuss it at an MDM and search for alternative options.

Another important finding was that care providers described the implementation of the law in home care settings as complicated and unclear, leading them to circumvent the law rather than adhere to it. According to the law, in cases of care‐resistant behaviour toward, e.g., tooth brushing and a found (risk of) serious harm, an MDM must occur with multiple care providers, including a doctor [7]. However, as our study reveals, it is often unclear for care providers involved in home care settings who this doctor should be: the general practitioner or the geriatric specialist. In addition, the general practitioners in our study all stated that they did not want to be the responsible party and were not present during MDMs. This finding is very much in line with an article from the Dutch Association for General Practitioners (LHV), in which they stated that involuntary care is not care fitting a general practitioner; general practitioners do not have a role in the application of or decisions about involuntary care at home [27]. Furthermore, a geriatric specialist is not routinely affiliated with home care organisations, making it unclear for care providers involved in home care who the responsible party is.

Another issue in home care settings, which can also apply to nursing homes, is that circumvention of the CCA implies that involuntary care or care similar to involuntary care may be provided without following the legal steps. For example, this occurs when medication is placed out of a patient's reach, or when care providers intentionally let the patient's family provide involuntary oral care as they do not have to follow the legal step‐by‐step procedure.

Both examples raise significant ethical concerns. Deliberately asking family members to provide involuntary care or circumventing legislation means that individuals with dementia are not rightfully protected, as the decision to provide such care is not regulated and carefully weighed among different care providers during an MDM. While not literally violating the law, caregivers in these examples clearly act against the spirit of the law, which may be regarded as ethically dubious. Possibly, they act this way for the sake of efficiency in care, as several participants found the CCA's step‐by‐step procedure unclear, and others considered it a large administrative burden. However, whether these are indeed the reasons motivating disrespect for legislation, and whether caregivers' weighting here is ethically acceptable, is a question that deserves more attention in future research.

Furthermore, our study shows that the CCA increased awareness of involuntary care, as the law requires care providers to follow a step‐by‐step procedure including an MDM to discuss whether it is really necessary to provide involuntary care. This increased awareness could reduce the unnecessary provision of involuntary care, as demonstrated by Bakkum et al. (2023) [28], who reported a decrease in registered cases of involuntary care after the CCA came into effect [28].

### 4.1 Strengths and Limitations

A strength of the present study is that it is, to the best of our knowledge, the first study to identify the experiences of care providers with legislation regulating involuntary oral care for individuals with dementia in the Netherlands. Identifying these experiences is important to understand the challenges concerning such laws and their implementation better. Based on this knowledge, tailored interventions and strategies can be developed to remove practical issues concerning legislation regulating involuntary oral care provision.

A limitation of the present study is that some participants described the behaviour or experiences of others regarding the legislation, which is hearsay and may be less reliable. Also, as this is a qualitative study, it does not quantify the frequency of certain experiences. Such quantifications would be valuable to provide a clearer understanding of the prevalence of specific challenges. Last, member checking was not performed due to practical concerns. While it might have strengthened the credibility of the results, other strategies, in particular peer debriefing, were used to support the trustworthiness of the findings.

## 5 Recommendations

1. Provide straightforward, practical examples of the legal concepts ‘care‐resistant behaviour’, ‘involuntary care’ and ‘serious harm’ in oral health care, using simple language to reduce differences in interpretation.
  - For instance, practical examples should include (1) patients verbally refusing oral care (such as saying ‘No’ once and afterwards allowing care, or consistently saying ‘No’), (2) patients non‐verbally refusing oral care by making bodily movements, and patients making bodily movements merely due to their illness, and (3) scenarios involving persuading (such as bribery, or distraction by use of music, TV‐programs, etc.).
2. Provide training on legislation and oral health care in both nursing homes and home care settings, and clarify which party holds responsibility in home care settings.
  - For instance, the training should address (1) the importance of oral care and how to provide it, (2) practical examples to understand the legal definitions, (3) dealing with care‐resistant behaviour in oral health care (searching for alternatives, which steps to undertake after failed attempts etc.), (4) the protocol of the (home) care organisation concerning involuntary oral care including a specification of individuals responsible for particular steps and (5) consequences of not providing oral care and behavioural changes caused by, for example, oral pain.

## 6 Conclusions

The present study identified experiences of care providers with legislation on involuntary oral care provision for individuals with dementia in the Netherlands. The CCA creates awareness about involuntary oral care among care providers. However, many care providers view the legal definitions differently, causing variation in the interpretations and applications of the legislation. Also, several care providers feel that the law could result in quicker discontinuation of oral care provision. Care organisations and policymakers should provide clarity concerning the legal definitions and provide training on legislation and the provision of oral health care in cases of care‐resistant behaviour.

## Author Contributions

Conceptualization, M.J., C.E. and A.V.; methodology, M.J., C.E. and A.V.; software, M.J.; validation, M.J. and C.E.; formal analysis, M.J. and C.E.; investigation, M.J.; resources, M.J. and A.V.; data curation, M.J.; writing – original draft preparation, M.J.; writing – review and editing, M.J., C.E., A.V. and D.J.M.; visualisation, M.J.; supervision, C.E. and A.V.; project administration, M.J. and A.V. All authors have read and agreed to the published version of the manuscript.

## Ethics Statement

The Medical Ethics Review Board (METc) of UMCG discussed the protocol and considered whether or not the research falls within the scope of the Medical Research Involving Human Subjects Act (WMO). The METc UMCG concluded that the protocol is not a clinical research study with human subjects as meant in the Medical Research Involving Human Subjects Act (WMO). Hence, the ethical review and approval were waived for this study (file number M24.326036).

## Consent

Informed consent was obtained from all subjects involved in the study.

## Conflicts of Interest

The authors declare no conflicts of interest.

## Data Availability

The data that support the findings of this study are available from the corresponding author upon reasonable request.
